# The RUMERTIME Process as a Protective Factor in School Attendance Problems

**DOI:** 10.5334/cie.40

**Published:** 2022-05-25

**Authors:** Yvonne Larrier, Monica Allen, Arline Edwards-Joseph, Geneva Fleming, Vanessa Kelleybrew

**Affiliations:** 1 Indiana University South Bend, US; 2 San Jose State University, US; 3 Liberty University, US; 4 Trident Technical College, US

**Keywords:** Cultivating SEEDS System®, RUMERTIME Process, social-emotional education, school attendance problems

## Abstract

The RUMERTIME Process (RP) is a five-step culturally responsive social-emotional, problem-solving, prevention-intervention strategy used to educate, equip, and empower students, educators, and families. The RP equips individuals with the abilities to recognize, understand, manage, express, and reflect on their thoughts, interactions, mindsets, and emotions (RUMERTIME) in relation to themselves, others, and the daily life challenges they face within multiple systems and settings.

The RP is embedded within the Cultivating SEEDS System framework (CSS) and is utilized to equip culturally diverse communities, inclusive of students, family members, educators, and administrators, with the social-emotional skills to effectively solve student attendance problems (SAPs). The data shared in this practice intervention article are descriptive in nature and highlight the RP as a protective factor and explain its three goals. The paper consists of three parts: (a) introduction of the RP, which is embedded in the CSS framework; (b) description of implementation of the RP as integral to the Daytime Intervention Room (DIR) program; and (c) discussion of risk factors that qualified students to receive services through the DIR program as well as data that demonstrated how the RP performed as a protective factor. The DIR program was aimed at creating an alternative to out-of-school suspension (OSS) and the traditional punitive in-school suspension (ISS). The program was established in each of the four schools in an urban high-needs school district in the midwest region of the United States. The DIR program was intentionally designed to include multiple levels, stakeholders, and delivery support, thus creating a solid base for the holistic development of students, educators, and parents. In conjunction with the CSS framework, the DIR program sought to increase academic performance, decrease the number of behavior referrals, and improve attendance rates in this high-needs urban school district.

## Introduction

Many factors keep students from attending school for prolonged periods of time. Physical illness is one such factor; others include lack of social-emotional wellness or mental ill health, such as a lack of self-awareness, lack of a sense of belonging, low levels of engagement, lack of caring adults, and lack of social-emotional competencies (SECs). Healthy social-emotional skills of caring adults can act as protective factors against stressful situations while promoting students’ social-emotional well-being and self-efficacy in the classroom (Arens & Morin, 2018; Poulou, 2018). When caring adults such as parents and school personnel are unable to effectively recognize, understand, and manage their own social-emotional health and wellness, these negative mindsets and emotions get transmitted to their children and students, which can result in consistent school withdrawal, a sense of disconnect from school, and a lack of belongingness. Consistent school withdrawal may also result in students experiencing psychological difficulties, including somatic illnesses, while being away from school for prolonged periods (Huber et al., 2019). Though these issues are not within the scope of this paper, their existence demonstrates why interventions like the RUMERTIME Process are integral to school and student success.

Teaching social-emotional skills to students and school personnel, as discussed in this practice intervention paper, acts as a protective factor by educating and equipping and empowering parents, educators, and students with the ability to collaborate on solving problems related to student attendance. When parents, educators, and students know how to respond, what to do, and where to go, harmful thinking patterns, mindsets, behaviors, and emotions are reduced, and students attend, and succeed in school. Efforts are made herein to offer a practice intervention of how the RUMERTIME Process works to help all stakeholders.

### Background

School attendance problems (SAPs) are considered a public health issue and a hidden educational crisis (Henderson et al., 2014; U.S. Department of Education, 2016), and have remained an area of concern in the United States for more than 100 years (Moehling, 2004). SAPs have deeply entwined roots and fruits like tentacles in multiple systems and segments of society. The multi-systemic nature of this long-standing crisis poses challenges for educators, administrators, policy, and decision-makers across diverse disciplines and segments of society with the adverse effects being felt far and wide (Kearney & Graczyk, 2020). Even prior to the recent COVID-19 pandemic, student attendance data were distressingly low, and this situation has not changed. For example, 1 out of every 6 students, 8 million students, or 15% of U.S. children, were missing three weeks or more of classes during the 2019–2020 school year. The students with the most absences belonged to historically underrepresented groups. These groups were hit hardest during the pandemic due to economic hardship, poor health, and unequal access to schooling (Chang et al., 2018). In 2020, one in four, or 25%, of students were unaccounted for nationally, and this number jumped to 40% in communities of color.

### Recognizing Student Attendance Problems (SAPs)

#### Elements of SAPs

APs are a complex, multifactorial issue that has attracted considerable attention from educators, policymakers, and practitioners both within and outside the field of education (Ekstrand 2015; Kearney, 2003). SAPs are enshrouded by myriad risk factors, including a lack of agreement about how to define, conceptualize, classify, assess, and address them. These complexities create implementation roadblocks for school administrators and educators at the school district and school building levels and generate dissemination and strategy development roadblocks for policymakers and researchers (Arora et al., 2016; Kearney, 2016, 2019). The urban midwest school district in which we implemented the RUMERTIME Process with the Daytime Intervention Room (DIR) program was experiencing these challenges at the school district and school building levels, hence the reason for engaging our services and utilizing the CSS framework as their prevention-intervention strategy.

### Social-Emotional Competencies (SEC)

Social and emotional skills are critical to being a good student, citizen, and worker (Collaborative for Academic, Social, and Emotional Learning [CASEL], 2021). Risky behaviors such as drug use, violence, bullying, absenteeism, and eventual dropout can be prevented or reduced when an ecosystemic, multiyear, integrated process is utilized to develop students’ social and emotional skills (CASEL, 2021; Christenson & Anderson, 2002; Innis, 2014; Schlund, 2021).

Some social-emotional learning (SEL) principles and programming have their genesis in Vygotsky’s sociocultural theory, in which he imports that human development occurs as a socially mediated process in which children acquire their cultural values, beliefs, and problem-solving strategies through collaborative dialogues or relationship capacity building experiences with more knowledgeable members of society (McLeod, 2018).

CASEL has identified five interrelated sets of cognitive, affective, and behavioral competencies: self-awareness, self-management, social awareness, relationship management skills, and responsible decision-making skills (CASEL, 2021). These five-core competencies are embedded in the RUMERTIME Process and are categorized as inter- and intrapersonal relationship capacity building skills (Larrier et al., 2017a). Research bears out that when students, families, and educators are educated, equipped, and empowered with these five social-emotional competencies from an ecosystemic perspective, students’ educational and life opportunities and outcomes are augmented (Beatty & Campbell-Evans, 2020; Catalano et al., 2002; Christenson & Anderson, 2002; DiPerna & Elliot, 2002; Schaps et al., 2004). Therefore, effective, holistic implementation of SEL has the potential to promote the academic, social, and emotional development of all children.

### The Cultivating SEEDS System (CSS) Framework

**Figure 1:**
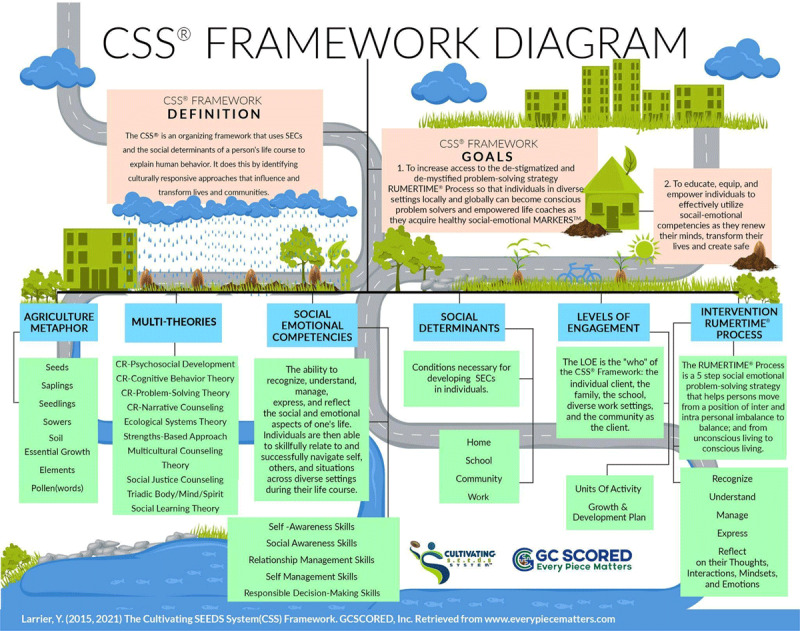
CSS Framework.

CSS is an organizing framework that gives context, order, and meaning to information obtained about a client’s background. A multitheoretical framework, it uses social-emotional competencies and the social determinants of a person’s life course to explain (why, what, and root) human behavior. CSS also identifies the culturally responsive approach of the RUMERTIME Process to educate, equip, and empower individuals as they renew their minds, transform lives, and create safer communities (Figure 1). CSS stands for the Cultivating SEEDS System. In turn, SEEDS is an acronym that stands for Social Emotional Education in Diverse Settings (Larrier et al., 2017b).

#### Goals of the CSS Framework

All CSS activities, training and coaching sessions, materials, and books are powered by CSS framework’s two underlying goals. The first goal is to increase access to the culturally responsive, destigmatized, and demystified problem-solving RUMERTIME Process so that individuals in diverse settings locally and globally can become conscious problem solvers and empowered life coaches as they acquire healthy social-emotional MARKERS. The second goal, intricately connected to the first, educates, equips, and empowers individuals to effectively utilize social-emotional competencies as they renew their minds, transform their lives, and create safer communities (Larrier et al., 2017b).

### The RUMERTIME Process

The RUMERTIME Process, the five-step culturally responsive, social-emotional, problem-solving strategy embedded in the CSS framework is a prevention-intervention strategy that moves individuals from a position of intra- and interpersonal imbalance to balance, and from unconscious to conscious living. Individuals of all ages are educated, equipped, and empowered with these skills to effectively navigate the daily difficulties and disruptions in their lives (Larrier et al., 2017a).

### The RUMERTIME Process as a Protective Factor

Within the developmental assets research community, protective factors are defined as skills, strengths, resources, supports, or coping strategies used by individuals, families, communities, or the larger society to help people deal more effectively with stressful events and mitigate or eliminate risk in homes, schools, and communities (Ozer et al., 2017). The RUMERTIME Process and rumerizing (processing) helps students address school attendance problems by first helping them to recognize and understand their thoughts (thinking patterns), interactions (behaviors), mindsets (beliefs), and emotions (feelings) about self, others, and possible environmental factor(s) impacting them and causing them to not attend, or stay in, school.

The step of recognizing and understanding thinking patterns, actions, beliefs, and feelings brings self-awareness skills into focus. Given their experiences at home, school, and in the community, students are taught systematically and developmentally what it means to be self-aware as they think about who they are, what they are good at, how they interact with their peers and adults, and what they believe and feel about school. As students acquire and practice the RUMERTIME Process in their daily activities, they learn to become conscious problem-solvers (Larrier et al., 2017).

According to Zins and Elias (2006), students who use problem-solving skills to rise above school challenges and make responsible decisions perform better academically. SEL programs like the RUMERTIME Process have been shown to have a positive impact on executive cognitive functioning (ECF) from early childhood to adolescence (Zins & Elias, 2007). ECF has critical implications for child and adolescent academic and social-emotional development, and is influenced by culture and adverse life events (e.g., poverty). ECF, specifically inhibitory control, trains children and adolescents to cope and manage by suppressing their maladaptive thoughts, behaviors, and emotions that influence environmental factors related to SAPs (Greenberg, 2006). This is where self-awareness skills and the ability to recognize and understand are seen as the most basic of the five core social-emotional competencies.

It is incumbent upon educators and schools who are utilizing SEL programming such as the RUMERTIME Process to ensure that the first two steps in their prevention-intervention strategies include self-awareness skills training. After foundations are laid with developmentally appropriate training in self-awareness skills and the ability to identify and understand maladaptive thoughts, interactions, mindsets, and emotions, students can then be educated and equipped to effectively incorporate other SECs such as self and relationship management, social-awareness, and responsible decision-making (Larrier & Kelleybrew, 2017c).

Once students are taught to assess their thinking patterns, behaviors, beliefs, and feelings honestly and accurately, they can become equipped to be active participants alongside socially emotionally balanced adults, who are empowered to effectively manage, express, and reflect on thoughts, interactions, mindsets, and emotions about various strategies to address their school attendance problems. The RUMERTIME Process systematically educates, equips, and empowers students, educators, and parents about the five core SECs. Additionally, the acquisition of these skills influences and impacts their abilities to cultivate supportive relationships and remain other-focused as they engage in empathic activities, while learning to make responsible choices during challenges associated with SAPs (Larrier et al., 2017a).

### Implementation of the RUMERTIME Process in an Inner-City School System

#### The Context

The DIR program was instituted by the newly hired school superintendent for the participating midsized school district in the midwest region of the U.S. The school district consisted of 99% Black students, and White teachers made up over 80% of the teaching staff. Over 75% of these white teachers lived outside of the community. The median district household income during implementation of the DIR program was $32,738. Sixty-four percent (64%) of the homes in this community were built before 1970; 64% had broadband internet service; 42% of the families lived below the poverty line, and 47% received food stamps and Supplemental Nutrition Assistance Program (SNAP) benefits; 53% of the households were female-led – no father was present in the household; 11.5% of the parents had a bachelor’s degree or higher. The student body at the beginning of the fall semester was 2,331: 1,234 males and 1,097 females.

### Program Design and Methodology

The RUMERTIME prevention-intervention specialists (RPISs) program was implemented as the SEL program to support the dynamic and innovative concept for the DIR project. The RPIS program, comprising multiple levels, stakeholders, and delivery supports, creates a solid base for the social-emotional development of participating students, educators, and parents. The goals of the collaboration were to decrease absenteeism, increase academic performance, and decrease behavioral referrals.

First, the RPIS training and coaching program will be described; next, the implementation of the RPIS program will be addressed.

### RPIS Training and Coaching

Participating RPISs received over 300 hours of training and coaching throughout the duration of the program, which lasted two full school years. These certified RPISs were the only ones in their respective schools to facilitate the sessions and lead in the DIR program. The training they received covered all the elements of the CSS framework, including the multiple underlying theories. Therefore, to provide support for the students referred to the DIR, an instructor had to be trained and coached in the CSS framework. Under no circumstances should the DIR program be facilitated by individuals without this intensive training of the CSS framework.

### Participants

Two weeks prior to the start of school, eight professionals were hired for RPIS training; they participated in 80 hours of the CSS framework training and coaching in preparation for utilizing and implementing their training in the innovative DIR program. Eighty percent (80%) of the trainees were female, and 20% were male. All of the trainees were people of color (POC). One hundred percent (100%) had a bachelor’s degree, 70% had a master’s degree, and 10% had a PhD. Their degrees were all related to areas in the fields of education, behavioral health, and human services. Further, 90% of the trainees resided within the school district community, and 100% of them had prior work experience in school systems and in youth development programs.

#### Recruitment of Participants

This position, titled intervention specialist, was advertised through the school district’s employment opportunity website portal.

#### Interview Process

The partner organization was asked to participate in the interviewing process after the minimum requirements screening was completed by the district human resources personnel. The partnering organization assumed leadership with regard to the development and delivery of the social-emotional case vignettes that were part of the interview process. The candidates had to submit their responses to the case vignettes in writing, and their responses were then used to evaluate their writing and comprehension skills. Additionally, candidates took part in a face-to-face interview in which they were asked to respond to school-based scenario type questions. Both nonverbal and verbal responses of the candidates were used to assess their abilities to respond under stressful situations.

## Implementation of the Intervention/Activities

### Educational Strategies and Materials

The RPIS training and coaching sessions utilized the *Sowers Path Facilitator’s Guidebook: From Root to Fruit* (Larrier, 2018). This guidebook contains all the necessary instructional tools, materials, and activities for the training (https://everypiecematters.com/products/category/books/). In addition, the trainer, who was the creator of this framework, was equipped with an assortment of CSS framework teaching and learning instructional tools such as card games, posters, PowerPoint lectures, utilized in the training. For the training and coaching sessions, staff utilized large-group sessions, small-group work groups, dyads, role play, and presentations by work groups and dyads.

### Program Delivery and Schedule

The 80 hours of RPIS training was delivered face-to-face in the school district’s central office conference room. The room was equipped with whiteboards for participant engagement and application; audio-visual equipment for presentations/teaching; small rooms for small-group work for role plays, sharing, and other small-group activities; flip charts and markers for sharing and large-group discussion and trainee presentations, and display boards for trainer and trainee artifacts. The training occurred over a two-week period, Monday to Friday from 8:00 a.m. to 5:00 p.m. with a one-hour lunch break. At the end of the 80 hours, the RPIS trainees received a certificate of completion and participation (Table 1).

**Table 1:** Program Schedule and Learning Objectives.


DAY	WEEK 1	DAY	WEEK 2

Day 1	Getting to Know Introduction to CSS Introduction to MARKERS™	Day 6	Manage (cope, handle) TIME-Self Management Skills

Day 2	RUMERTIME Process Multitheories Social-emotional competencies	Day 7	Manage (influence, inspire) TIME-Relationship Management Skills

Day 3	Recognize (identify, list) TIME-Self-Awareness Skills	Day 8	Express (other-focused, empathy) TIME-Social-Awareness Skills

Day 4	Understand (dig deeper) TIME-Self-Awareness Skills	Day 9	Reflect (choices, reflection) TIME-Responsible Decision-Making Skills

Day 5	Understand (dig deeper) TIME-Self-Awareness Skills	Day 10	Connections & Transformations TIME


Attendance and participation was mandatory for RPIS trainees, who each received a stipend to attend the two-week 80-hour training.

### Evaluation

A 10-item pre-/post-training evaluation was conducted. The first eight items were posed in a 5-point Likert scale format (e.g.,*“The RUMERTIME Process moves a person from a place of imbalance to balance.”*). Questions 9 and 10 were fill-in-the-blank type items (e.g., *“List three benefits from learning and practicing the SECs.”*).

The pretraining survey was administered as the post-training evaluation. At the end of each day, RPIS trainees were required to engage in a 10-minute reflection. Here are comments from two trainees, *“I’ve never been to such an intense training session; this is like therapy.” “This training is getting to the root, I love it.”*

### Coaching Sessions

The coaching component of the training officially commenced once the students returned to the buildings for the fall semester. Once the trainers had completed the official 80-hour training sessions prior to the start of the fall semester, they transitioned to providing in-person coaching for certified RPISs twice a month. There were two RPISs for each building: kindergarten-2^nd^ grade; 3^rd^–5^th^ grade; 6^th^–8^th^ grade; and 9^th^–12^th^ grade. CSS toolkits were made available to the RPISs – one toolkit per DIR. Each toolkit contained instructional aids, such as posters, facilitator guides, student workbooks, games, activity sheets, and instructions for data collection. RPISs were given permission to make unlimited copies of the activity sheets and workbooks.

## Results

The scores from the pre- and post-assessments were calculated using frequency statistics and percent change. The results (see Table 2) showed that the trainees’ scores increased. To measure the amount of growth, we calculated the percentage of change between the two sets of scores.

**Table 2:** Pre- and Post-Test Scores.


QUESTIONS	PRE-TEST	POST-TEST	PERCENTAGE CHANGE

Question 1	2.75	5.00	81%

Question 2	1.00	1.00	No increase

Question 3	3.50	5.00	43%

Question 4	2.62	5.00	91%

Question 5	3.13	5.00	51%

Question 6	3.00	5.00	67%

Question 7	2.90	5.00	72%

Question 8	3.00	5.00	67%

Question 9			70%

Question 10			100%


### Implementation of the RUMERTIME Process

#### Student Participants

At the time of the DIR program implementation, the total number of students enrolled in the participating school district was 2,331: 1,097 females and 1,234 males. The number of students who were referred to the DIR during the two-year period was approximately 1,038: 406 were females and 632 were males. Table 3 gives a breakdown of the number of students served by grade level and gender, as well as reasons for visits. The “Other Referrals” included other types of referrals such as referrals for disruptive behaviors in classrooms, bullying, teacher-student conflict, emotional outbursts, and so on.

**Table 3:** Students Served by Gender, Grade Level, and Reason for Visit.


ITEMS	K-2^nd^	3^rd^–5^th^	6^th^–8^th^	9^th^–12^th^

Females	126	62	140	78

Males	164	158	182	128

Total	290	220	322	206

Other Referrals	170	120	250	28

Suspensions	120	100	72	178


#### Setting

Several of the buildings in the school district were old and under-resourced. For example, the high school is 100 years old and in a state of disrepair. In addition, the resources, such as books, technology, and other learning tools, were outdated and unusable. Over half of the students lived with one parent (mothers). Over 90% of the students were in general education classes but performed below grade level in reading and math.

#### Environment

The RPISs were allocated classrooms in each building that were not otherwise being utilized at the time. Each dyad came together and decorated their DIR space utilizing many of the CSS framework posters and other artifacts to display on the walls. Each RPIS dyad planned different themes and layouts for their space depending on the age group with whom they worked. However, each DIR had an inviting, relaxing, non-threatening atmosphere. Many of them had couches, round tables, and chairs; none of these rooms resembled a typical classroom setup.

### Learning Objectives for Students

Social-Emotional Wellness according to the CSS framework refers to a person’s ability to **R**ecognize, **U**nderstand, **M**anage, **E**xpress and **R**eflect on their **T**houghts, **I**nteractions, **M**indsets and **E**motions (**RUMERTIME®**) as it relates to self, others, and situations. Effectively utilizing the RUMERTIME Process educates, equips, and empowers individuals of all ages, stages, and locations to successfully navigate daily activities such as loving, learning, earning, relationship building, problem-solving, and adapting to the demands of growth and development (Larrier et al., 2017a).

The activities that students engaged in when they came into the DIR were grounded in the following standards/outcomes:

- Ability to Recognize (identify/Self-Awareness Skills) their Thoughts (Ideas), Interactions (behaviors), Mindsets (beliefs), and Emotions (feelings) about themselves, others, and the SAPs for which they were referred to the DIR.
- Ability to Understand (dig deeper/Self-Awareness Skills) their Thoughts (Ideas), Interactions (behaviors), Mindsets (beliefs), and Emotions (feelings) about themselves, others, and the SAPs for which they were referred to the DIR.
- Ability to Manage (Cope/Self-Management and Relationship Management Skills) their Thoughts (Ideas), Interactions (behaviors), Mindsets (beliefs), and Emotions (feelings) about themselves, others, and the SAPs for which they were referred to the DIR.
- Ability to Express (Other-focused/empathy/Social-Awareness Skills) their Thoughts (Ideas), Interactions (behaviors), Mindsets (beliefs), and Emotions (feelings), about themselves, others, and the SAPs for which they were referred to the DIR.
- Ability to Reflect (Evaluation/Responsible Decision-Making Skills) their Thoughts (Ideas), Interactions (behaviors), Mindsets (beliefs), and Emotions (feelings) about themselves, others, and the SAPs for which they were referred to the DIR.

### Materials

Multiple activities were used for the various interactions in the DIR. For example, hanging on the entrance to each DIR was a RUMERTIME Emometer poster, where students could assess their emotional status upon entering and leaving the DIR. Additionally, each RPIS had a Growth and Development Plan (GDP) book for each student. Additionally, a wide range of materials were used depending on the emotional state in which the student came into the DIR; for example, one RPIS found that puzzles worked well to calm down some of her middle school students so that they could then process (rumerize) the SAP which they had been referred to the DIR. Journaling was also used on a regular basis with many of the students. Finally, the RUMERTIME Guided Activity Journal (RGAJ), a multimodal journal, allowed students to choose whether they wanted to write, draw, color, or engage in a brain teaser as a form of self-expression and self-management. Many of the male students gravitated to drawing as a form of expressing their thoughts, interactions, mindsets, and emotions.

## Implementation of the Intervention/Activities

### Educational Strategies

The RUMERTIME Process in the DIR was delivered primarily (70%) as individual sessions, secondarily (20%) in the form of small groups, and 10% of the time was spent in the classroom teaching social-emotional competency lessons. The individual sessions were initiated by the referrals that flowed from the teachers to the school’s administrative team and then to the RPIS.

#### The Growth and Development Plan (GDP) Book

The GDP book was designed with a comprehensive biopsychosocial model in mind, like the CSS framework and utilizes the multiple theories embedded in the CSS framework®. As such, it provided the RPIS with an ongoing intake/progress notes/intervention tool that helped guide and structure the interactions between the RPIS and the student from the first to the final session. For example, it included questions and other content that the RPIS could bring up with the student. The questions were divided into categories based on the theories and the ecosystems. It also provided opportunities for the RPIS to consolidate the information about the student, giving the RPIS specific and global details about their students. The information gathered in the GDP as is stated in the definition of the CSS framework organizes and gives meaning, order, and context to an individual’s background.

#### Narrative Counseling – The RUMERTIME Storyboard Book

The RUMERTIME Storyboard Book was another educational strategy with which students were allowed to share their experience, as if they were the narrator of a story from at least three perspectives, including their own. The RUMERTIME Storyboard book is one of the 25 CSS framework activities and instructional tools designed around the CSS framework. As mentioned, the CSS is a multitheoretical framework, including narrative counseling theory. Narrative counseling is an intervention strategy that separates students from their problem and encourages them to recognize and understand their skills to manage the problems in their lives. Students’ personal experiences, whether at home, school, or in the community, become their personal stories; they give these stories meaning, and the stories then help to shape their identity.

Narrative counseling as an educational strategy embedded in the RUMERTIME Storyboard Book empowers students to use their stories to help them see their problems within a larger sociocultural context, educates them on how to make space/room for other stories, equips them with the ability to find a connection between their actions and choices, and helps them to understand at a deeper level how they experience life and thereby gain agency for problem-solving in the future.

### Program Delivery and Schedule

Students who were referred to the DIR by the administration were automatically added to the RPIS caseload. However, this did not prohibit students who were in crisis situations to drop in as needed with permission from a teacher or administrator; in fact, many times these students who were walk-ins remained on the RPIS caseloads. Students were referred to the DIR in their building as the last stop before being sent to ISS or OSS.

The DIR spaces were designed to educate, equip, and empower the referred student to be able to recognize, understand, manage, express, and reflect on their thoughts, interactions, mindsets, and emotions in relation to the challenges and problems they were experiencing, often showing up as attendance problems, maladaptive behaviors, and poor academic performance. If a student received OSS, the student and their parent were required to meet with the RPIS at the student’s school in the DIR before coming back into school after the OSS, to introduce the parent to the RUMERTIME Process and develop an Individualized RUMERTIME Prevention Intervention Plan with the student and the parent to reduce the likelihood of the student reoffending. The RPISs had access to student records and relevant teachers and administrators so that they could create a safety net for the students to be successful.

Student sessions typically lasted from 15 minutes to more than 60 minutes. Length of the sessions was dependent on many factors, primarily the emotion intensity level of the student entering the DIR. The RUMERTIME intervention tool/strategy that was used during the session was a collaborative decision between the RPIS and the student. Because the RPISs were extensively trained in the effective utilization and implementation of the CSS framework and its tools, they were able to accurately assess the needs of the students and hence apply the relevant tools to help move the students from inter- and intrapersonal imbalance to balance.

### Evaluation

Multiple evaluation tools were used, such as the RUMERTIME Emometer as well as summative and formative qualitative feedback obtained from open-ended questions given to students, RPISs, teachers, administrators, and parents. These were all administered by the RPISs on one level and then by the trainers on another level of evaluation. Additionally, RPISs were required to complete GCSCORED’S daily interaction logs developed specifically for the DIR program. The GCSCORED interaction logs contained 22 questions; developed in SurveyMonkey, the logs were accessible to each RPIS using a customized link. Each student interaction was logged as an individual entry.

## Results

### Methods

A total of eight RPISs entered data reflecting 1,174 interactions. The student data focused on grade, grade-level groups, reasons for visits to the DIR, and use of the RUMERTIME Process. The data were prepared in Excel for analysis; analysis was done using SPSS, and open-ended questions were analyzed using NVivo. Additionally, the two high school RPISs conducted a case study with five male students with chronic discipline referral issues. They worked with these five students in the fall of 2016 until the end of the 2018 school year. (These data are presented and explained in this paper.)

### Reasons for Visiting DIR

The RPISs were asked *“Why did the student come to see you? Please select all that apply.”* There were five response options, “*1. possible suspension, 2. suspension, 3. possible attendance issues, 4. possible academic issues and 5. other.”* The respondents were able to indicate multiple reasons which is why the overall percentage does not add up to 100%. The large majority chose *“Other”* (85%). The second most frequently mentioned reason was *“possible suspension”* (11%), followed by *“possible academic issues”* (7%), *“possible attendance issues”* (4%), and *“suspended”* (4%). Respondents who indicated *“Other”* were asked to specify their choice. There were 993 responses to this question. These 993 open-ended responses were analyzed in Excel. A new column was created showing the theme(s) touched upon in the responses. When answers fell in more than one category, double-coding was allowed. This meant that when more than one theme was mentioned, the answer was coded with multiple themes. Therefore, in total there were more mentions of themes than number of responses. As shown in Table 4, 38% of the 993 entries involved “Check-In/Monitoring/Caseload/Observation.” The second most mentioned reasons were behavioral issues, which included defiant behaviors, challenging authority, not following rules, altercations, and anger issues. Another large group of responses involved bullying, being bullied, fighting, or simply not knowing how to manage peer relationships as problematic. Social-emotional issues were mentioned almost 1 in 10 times and included anxiety, lack of self-management, frustration, crying, and grief, among others.

**Table 4:** Reasons for Visiting DIR.


REASONS – OTHER	NUMBER OF MENTIONS	PERCENT OF TOTAL RESPONSES

**Check-In/Monitoring/Caseload/Observation**	**381**	**38%**

**Behavioral Issues**	**214**	**22%**

**Interactions With Other Students**	**129**	**13%**

**Social-Emotional Issues**	**85**	**9%**

**Classroom Behavior**	**76**	**8%**

**RUMERTIME**	**45**	**5%**

**Absenteeism/Tardiness**	**22**	**2%**

**Relationship Management**	**15**	**2%**

**Support/Supervision**	**14**	**1%**

**Academic Issues**	**9**	**1%**

**Referral**	**8**	**1%**

**Other**	**48**	**5%**


### Attendance

**Figure 2:**
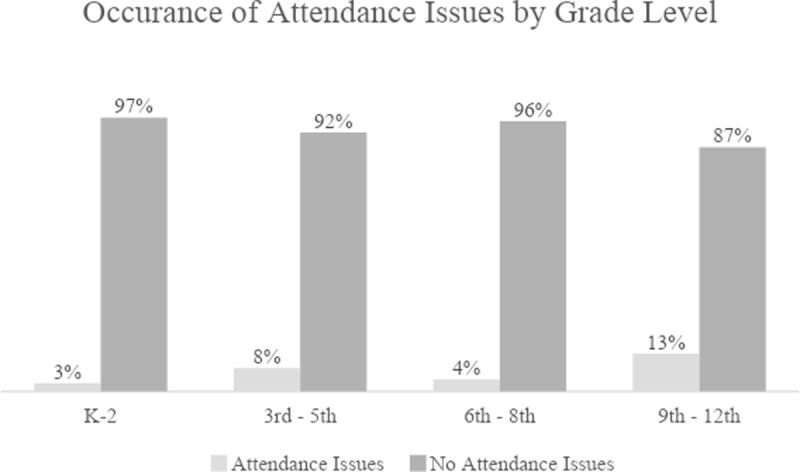
Occurrence of Attendance Issues by Grade Level.

One of the questions on the interaction log was *“Why did the student come to see you?”* Here the RPISs were to choose all the applicable response options.” There were five multiple-choice response options, such as *“1. possible suspension, 2. suspended, 3. possible attendance issues, 4. possible academic issues, and 5. Other.”* Some of the respondents who chose *“Other,”* also included attendance-related issues. Furthermore, if respondents indicated the student was in danger of being suspended, they were asked to state the reason for the possible suspension. Many mentioned attendance issues here as well. When taken together, attendance came up as an issue in 89 of the 1,174 responses where these questions were answered (8%). As Table 5 and Figure 2 show, attendance issues tended to be almost *“no attendance issues”* in K-2, but much more common in Grades 9 through 12.

**Table 5:** Attendance.


	K-2	3^rd^–5^th^	6^th^–8^th^	9^th^–12^th^

**Attendance Issues**	**8**	**16**	**14**	**51**

**No Attendance Issues**	**265**	**178**	**314**	**328**

**Total**	**273**	**194**	**328**	**379**


School attendance is critical if students are to experience school success and acquire the social-emotional protective factors necessary to navigate K-12 and become productive citizens. Elias and Haynes (2008) suggested that for K-12 students, especially students from lower socioeconomic backgrounds, acquisition of SECs serves as a protective factor. Similarly, a meta-analysis by Durlak et al. (2011) on SECs showed that SEL programming such as the RUMERTIME Process demonstrated gains in social-emotional skills and academic achievement, and improved behavior at school.

### Suspensions

The RPISs were asked *“Is this student in danger of being suspended?”* Response options were *“Yes”* and *“No.”* The large majority chose *“No”* (77%). RPISs who indicated that the student was *“in danger of being suspended”* were asked a follow-up question, *“Why was the student in danger of being suspended?”* There were 259 responses related to this “why” question. The 258 open-ended responses were analyzed in Excel. A column was created for each theme touched upon in the responses. When answers fell in more than one category, double coding was allowed. This meant that when more than one theme was mentioned, the answer was coded with multiple themes. Therefore, in total, there were more mentions of themes than number of responses. As shown in Table 6, 50% of the 258 entries highlighted behavioral issues as reasons for students being in danger of suspension (e.g., behavioral issues such as defiant behaviors, challenging authority, not following rules, and aggressive and destructive behaviors). Another cluster of responses related to student peer interactions involved arguing, fighting, or intimidating other students (44%). Classroom behavior was the problem in 13% of the cases and consisted mostly of disrupting the classroom. Finally, attendance issues was another problematic area, which included mostly skipping class and elopement (12%).

**Table 6:** Reasons for Suspension.


REASON – DANGER OF SUSPENSION	NUMBER OF MENTIONS	PERCENT OF TOTAL RESPONSES

**Behavioral Issues**	**129**	**50%**

**Interactions With Other Students**	**113**	**44%**

**Classroom Behavior**	**34**	**13%**

**Attendance Issues**	**31**	**12%**

**Other**	**3**	**1%**

**Social-Emotional Issues**	**1**	**0%**


Research conducted by Greenberg et al. (2003) highlighted that when students do not possess SECs, negative student outcomes are evident. In other words, at the root of low academic performance, poor grades and overall achievement, discipline referrals, poor attendance and dropout rates are deficits in students’ social-emotional skills. Furthermore, students who had limited to no social-emotional skills were also more likely to have a higher number of disciplinary referrals and/or be referred for special education due to problematic behaviors (Elias & Haynes, 2008; Stoiber, 2011).

Even though Table 6 shows that there was only one mention of social-emotional issues, given the research discussed earlier (Elias & Haynes, 2008; Greenberg et al., 2003; Stoiber, 2011), at the core of the reasons for *“danger of suspension”* was a lack of, or limited, social-emotional competencies in students. When students acquire SECs, it creates strong roots and sturdy trunks, which act as foundational, protective, and supportive factors in students’ growth and development.

### High School RPISs Case Study

**Figure 3:**
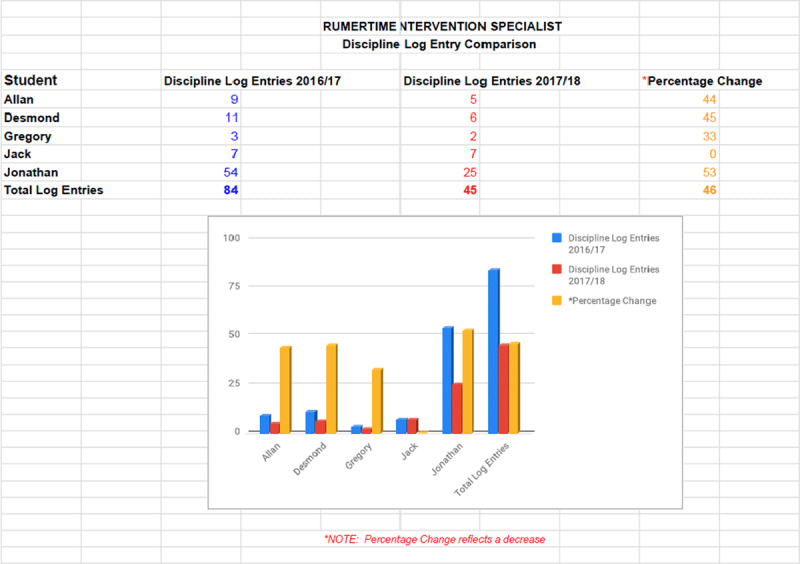
RPIS Discipline Log Entry Comparison.

Figure 3 shows the results of a group of high school males who were on the caseload of the RPISs and were followed from fall 2016 to June 2018. (Names were changed for anonymity.) The RPISs utilized a variety of CSS framework tools and techniques during this time with these five males.

As the RPISs implemented the RUMERTIME Process with fidelity, a substantial positive change was seen in the discipline referrals for the five high school males. These results are undergirded by the social-emotional learning (SEL) research, which suggests that when schools implement, with fidelity, SEL programs such as the RUMERTIME Process, student conduct problems, including alcohol and drug use, dropout, and non-attendance, decrease and SECs, attitudes and positive behaviors, improve (Durlak et al., 2010; Taylor et al., 2017; Wilson et al., 2001).

Other research suggests that low achievement and chronic absenteeism (Schnittka-Hoskins, 2021) cause sharp declines in students’ self-awareness, efficacy, self-management, social-awareness, and engagement. According to Schnittka-Hoskins (2021), the SECs that are most linked to issues surrounding absenteeism include self-management, self-efficacy/awareness, social awareness, and growth mindset/engagement, suggesting that student absenteeism adversely impacts all four SECs. Schnittka-Hoskins (2021) also showed that as students’ absences increased, their social awareness skills declined. Furthermore, absences had about equal adverse effects across all student subgroups. For example, elementary students with extended absences demonstrated a decrease in self-efficacy and self-management skills. Middle school students with extended absences demonstrated the strongest decline in self-efficacy and social awareness skills, whereas high school students with extended absences demonstrated the strongest decline in social awareness skills. Overall, elementary and middle school students suffered the greatest social-emotional disruption and decline when experiencing extended absences (Schnittka-Hoskins, 2021).

### Outcomes of RUMERTIME Intervention Strategies

#### RUMERTIME Emometer

**Figure 4:**
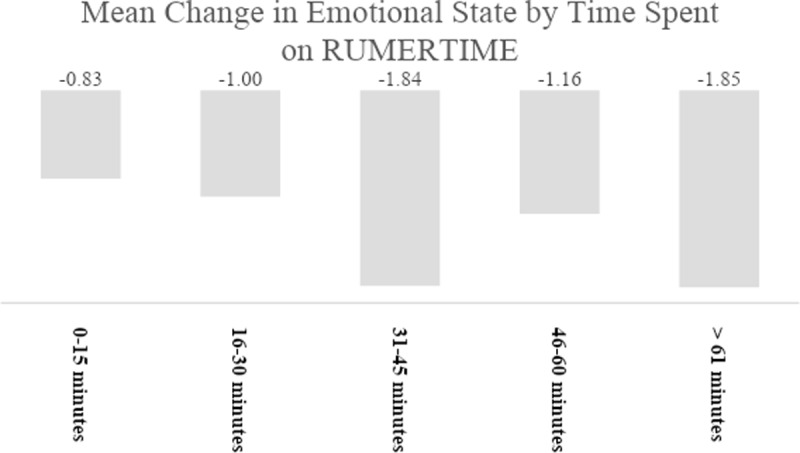
Mean Change in Emotional State by Time Spent on RUMERTIME.

As part of the DIR programming, every time a student entered and exited the DIR, the RPISs were required to assess the students’ emotional state. Specifically, they asked students to rate their emotional state on a scale of 1–10 (1 being low intensity and 10 being high-intensity emotion). A total of 810 interactions were rated for these two variables by the RPICs. The average change was –1.40. The results of a paired *t*-test used to compare the two values showed that the difference was highly statistically significant (*p* < 0.001). The emotional state when the students left was significantly lower (3.39 out of 10) than when the students came in (4.79 out of 10) (Figure 4).

Utilization of the RUMERTIME Emometer was an integral part of the RP and is grounded in research while supporting our assumption that there is a connection between students’ ability to recognize, understand, manage, express, and reflect on their emotions and academic tasks, levels of school engagement, relationships with peers and adults, general academic achievement/performance, and SAPs. According to Reschly et al. (2008), increased positive emotions are correlated with higher levels of student engagement, while negative emotions are associated with lower levels of engagement. Further, Papousek et al. (2010) posited that **s**tudents who experience more positive emotions throughout the school day may demonstrate greater management skills and be better able to recover from test anxiety and other anxiety generating activities. Suldo et al. (2013) also suggested that as the intensity levels of positive emotions increases, there is a direct, predictive relationship with math and reading grades two years later in adolescents. Um and colleagues (2007) found that this relationship shows up in elementary school students as high levels of cognitive investment and satisfaction. In short, the statistically significant results of RUMERTIME Emometer activity supports the idea that recognizing and understanding students’ emotional states in the classroom helps to indirectly and, in some cases, directly eliminate barriers to academic success, non-attendance and SAPs, and create optimal learning spaces.

### Time Spent Using the RUMERTIME Process

The RPISs were also asked how much time they spent using the RUMERTIME Process. The times ranged widely, from 0–15 minutes to over 61 minutes. Differences between the group for mean change in emotional state were tested using ANOVA, which showed that at least one group was significantly different from another (*p* = .005). Post-hoc analyses (Bonferroni) indicated that the mean change in emotional state was significantly higher when the time spent on the RUMERTIME® was more than one hour compared to only 16 to 30 minutes (*p* = .040) (Table 7).

**Table 7:** Time Spent on RUMERTIME® and Change in Emotional State.


	MEAN CHANGE IN EMOTIONAL STATE	*n*	STANDARD DEVIATION

**0–15 minutes**	**–0.83**	**88**	**2.39**

**16–30 minutes**	**–1.00**	**204**	**2.38**

**31–45 minutes**	**–1.84**	**142**	**3.19**

**46–60 minutes**	**–1.16**	**142**	**3.09**

**> 61 minutes**	**–1.85**	**231**	**3.71**


## Conclusion

Protective factors are skills, strengths, resources, supports, or coping strategies in individuals, homes, schools, communities, or the larger society that help people manage more effectively stressful and traumatic events in diverse settings (Howard et al., 2010). The outcomes presented here suggest that the RUMERTIME Process intervention strategy utilized by the RPISs during the students’ time in the DIR strongly influenced their emotional state in a positive way; for an example, please review this YouTube Recording of the RPIS and students in the DIR Program.

When students can recognize, understand, and manage their emotions, they are better able to show empathy and engage in instructional time, their attendance improves, discipline referrals decrease, and academic performance increases. They become socially aware and are better able to make responsible decisions/choices.

As students, educators, family members, and other adult role models acquire the skills taught in the RUMERTIME Process, their ability to effectively relate to self and others increases to the level of a protective factor. Additionally, their ability to successfully navigate the daily life challenges surrounding school attendance while showing up and engaging at school despite their lived realities suggests that the RUMERTIME Process has some level of influence on their mindset, activities, relationships, knowledge, emotional literacy skills, resources, and strategies.

### Future Directions

When students attend a school where they feel valued and like they matter, the attendance crisis will begin to abate. Therefore, we propose that all schools, especially urban high-poverty, high-needs school districts like the one discussed in this paper, consider a prevention intervention program like the RUMERTIME Process and adopt a new way of working with schools, families, and communities struggling with SAPs.
